# Novel features of Mycoplasma genitalium genomes identified through Oxford Nanopore sequence analysis of isolates from Australia

**DOI:** 10.1099/mgen.0.001622

**Published:** 2026-01-28

**Authors:** Jose L. Huaman, Catriona S. Bradshaw, Teck-Phui Chua, Erica L. Plummer, Jennifer A. Danielewski, Lenka A. Vodstrcil, Suzanne M. Garland, Gerald L. Murray

**Affiliations:** 1 Department of Obstetrics, Gynaecology and Newborn Health, University of Melbourne, Parkville, Victoria, Australia; 2 Centre for Women’s Infectious Diseases, The Royal Women’s Hospital, Parkville, Victoria, Australia; 3 Molecular Microbiology Research Group, Murdoch Children’s Research Institute, Parkville, Victoria, Australia; 4 Melbourne Sexual Health Centre, Alfred Health, Carlton, Victoria, Australia; 5 School of Translational Medicine, Monash University, Melbourne, Victoria, Australia; 6 Centre for Epidemiology and Biostatistics, Melbourne School of Population and Global Health, University of Melbourne, Parkville, Victoria, Australia

**Keywords:** dual-class resistance, Genome Sequence, *mgpB*, *Mycoplasma genitalium*, Nanopore, translocation

## Abstract

*Mycoplasma genitalium* is a fastidious human pathogen with increasing antimicrobial resistance, yet its genomic landscape remains poorly characterized due to difficulties with culture, including prolonged incubation periods and low DNA yields from both clinical and cultured samples. Consequently, there are few publicly available genome sequences. In this study, a Vero cell culture protocol was optimized to increase *M. genitalium* DNA yield and integrated with Oxford Nanopore technology. As a result, 22 complete genome sequences were generated with a mean sequencing depth of 51.01×. Comparative genomics revealed that 59% of isolates contained a translocated rRNA operon, with junction flanks showing ~90% identity to the *MgPar* repetitive regions known to be associated with genomic rearrangement. Phylogenetic analysis revealed multiple groups encompassing both recent and deeply branching lineages. High rates of macrolide (90.9%) and fluoroquinolone (45.5%) resistance were observed. All isolates with quinolone resistance mutations also carried macrolide resistance mutations. Notably, all three isolates with *mgpB* 161 allele had the same resistance profile: A2059G, H69R, S83I and M95I at 23S, L4, *parC* and *gyrA*, respectively. This work provides the first complete *M. genitalium* genome generated using Oxford Nanopore sequencing from Vero cell-propagated isolates, underscoring the novelty and technical advancement of this approach.

**Impact Statement**

There are very few published *Mycoplasma genitalium* genomes, hindering research into this sexually transmitted pathogen. Our study presents methodology for *M. genitalium* culture, sequencing and analysis using the Oxford Nanopore Technologies MinION system, highlighting challenges and solutions. Twenty-two new genome sequences are presented, corresponding to recent isolates.

## Data Summary

Complete genome sequences generated in this study are available in the GenBank database, BioProject PRNJNA1326634 (Table S2, available in the online Supplementary Material). Additionally, supplementary material has been published on figshare (https://doi.org/10.6084/m9.figshare.31062259)

## Introduction

*Mycoplasma genitalium* is a sexually transmitted pathogen recognized for its role in urogenital tract infections in men and women [1,2] and for its increasing resistance to key antimicrobial agents [3]. Resistance to macrolides, a primary treatment option, is driven by SNPs at positions 2058 and 2059 in the 23S rRNA gene (*Escherichia coli* numbering) [4]. Similarly, fluoroquinolone resistance arises from amino acid substitutions within the quinolone resistance-determining regions (QRDRs) of the *parC* and *gyrA* genes, such as S83I in *parC* and M95I in *gyrA*. These mutations reduce drug affinity for DNA gyrase and topoisomerase IV, compromising treatment effectiveness [5].

Despite significant advances in identifying resistance-associated mutations in *M. genitalium*, our understanding of its broader genomic landscape, including recombination events and specific genetic alterations beyond known resistance markers, remains limited. This knowledge gap primarily arises from technical challenges in obtaining sufficient high-quality DNA for genome sequencing. *M. genitalium* is notoriously difficult to isolate and culture, often requiring extended incubation periods of up to several weeks [6]. Moreover, direct sequencing from clinical samples is impractical due to the low bacterial load in infections [7,9]. These factors collectively pose substantial challenges in generating sufficient DNA *in vitro* for sequencing purposes, thereby limiting our understanding of *M. genitalium* genomic complexity and evolution.

The complete genome of *M. genitalium* was first published in 1995, revealing that it possesses the smallest known genome (0.58 Mb) among free-living organisms, with fewer than 500 predicted protein-coding genes [10]. Although sequencing technologies have advanced considerably in recent years, the number of *M. genitalium* complete genomes available in public databases remains relatively low compared with other sexually transmitted pathogens. This scarcity restricts comparative genomic analyses and the understanding of population-level diversity. Notably, Fookes *et al*. [11] examined temporally and geographically diverse isolates and revealed a highly conserved core genome with no accessory regions, alongside extensive homologous recombination across 25 genomic loci [11]. Such recombination appeared to be mediated by a system of homologous exchange in which genetic information is transferred between repetitive elements, consistent with mechanisms previously described for variation at the *MgPar* loci [11,13].

Herein, we present a pipeline for sequencing cultured *M. genitalium* isolates using the Oxford Nanopore Technologies (ONT) platform. By optimizing a Vero cell culture protocol and integrating with long-read sequencing, we reliably produced high-quality, complete *M. genitalium* genome sequences.

## Methods

### *M. genitalium* isolates and culture methods

*M. genitalium* isolates were obtained from samples collected at the Melbourne Sexual Health Centre between September 2022 and October 2024, from the ‘MAGIC’ study described previously [6]. Ethics approval was obtained from the Alfred Hospital Ethics Committee (project number 262/15). Bacteria were isolated from urine and high vaginal swabs through Vero cell co-culture and identified by quantitative PCR (qPCR), as previously reported [6].

In the present study, we adapted a previously reported methodology [6] to generate a sustainable supply of *M. genitalium* DNA for genomic analysis. Vero cell monolayers at 70% confluence were inoculated with ~10^6^ genome equivalents (geq) of *M. genitalium* and maintained in Eagle’s Minimum Essential Medium (Sigma-Aldrich, MO, USA; Cat# M5650-500ML) supplemented with 2% Ultroser G serum substitute (Sartorius, Cergy-Saint-Christophe, France) and 1% GlutaMAX™ Supplement (Gibco, Thermo Fisher Scientific, Waltham, MA, USA). Cultures were incubated at 37 °C and 5% CO_2_, and culture media were replaced 7 days post-infection and subsequently at weekly intervals. Monolayer health and confluence were assessed microscopically each week, with fresh Vero cells added when confluence dropped below 70%. Growth was monitored by qPCR targeting the *mgpB* gene [14].

### DNA extraction

DNA extraction used approaches designed to minimize shearing of genomic DNA (gDNA) to capitalize on the long-read potential of ONT sequencing. Four millilitres of culture with *M. genitalium* loads ranging between 10^7^ and 10^8^ geq ml^−1^ were centrifuged at 20,000 ***g*** for 30 min at 4 °C. The supernatant was discarded, and the pellets were resuspended in 1X DNA/RNA Shield (Zymo Research, Irvine, USA). gDNA was extracted using the Quick-DNA HMW MagBead Kit (Zymo Research) by following the manufacturer’s instruction. The DNA yield was quantified using the Qubit™ dsDNA High Sensitivity Kit (Thermo Scientific, #Q32853) and stored at −80 °C until further analysis. Bacterial content was quantified by qPCR as described previously [14].

### Nanopore sequencing and genome assembly

Sequencing libraries were prepared from 400 ng of gDNA using the Ligation Sequencing Kit SQK-NBD114.96 (Oxford Nanopore Technologies, Cambridge, UK) and sequenced on a MinION Mk1C device with a FLO-MIN 114 R10.4.1 flow cell (Oxford Nanopore Technologies). Five to ten samples (depending on the number of pores available) were multiplexed per flow cell to optimize the sequencing. An initial 24-h sequencing run was followed by DNase treatment to remove residual DNA, after which the same library was reloaded and sequenced for an additional 24 h.

Basecalling, adapter trimming and demultiplexing were performed using Dorado v0.7.2 and the super high accuracy model (dna_r10.4.1_e8.2_400bps_sup@v4.2.0). Reads with a read quality below 9 and shorter than 1,000 bp were filtered using Nanoq v0.10.0. Coverage and sequence depth were calculated using Minimap2 v2.26 [15] and SAMtools v1.16.1 [16].

*De novo* assembly was conducted using Flye v2.9.2 [17], based on an estimated genome size of 0.6 Mbp for *M. genitalium* G37 (GenBank accession no. NC_000908). Medaka v1.9.1 (https://github.com/nanoporetech/medaka) using the model r1041_e82_400bps_sup_v4.2.0 was applied for assembly polishing. A minimum sequencing depth of 30× was set for assembly. If this threshold was not met, a second sequencing run was conducted, and reads from both runs were merged prior to assembly. Final assemblies were manually inspected using Geneious Prime version 2025.0.2 (Biomatters Ltd, Auckland, New Zealand). Complete genome sequences were annotated based on the *M. genitalium* G37 reference strain (NC_000908) and extracted using Geneious Prime using default settings. Gene synteny was evaluated using the ‘progressiveMauve’ algorithm with default parameters [18]. The assessment of genome assembly and sequence contamination annotation completeness was calculated using BUSCO v5.4.5 [19] against the Mycoplasmatales odb10 database. *mgpB* typing was determined via the PubMLST *M. genitalium* typing scheme (https://pubmlst.org/organisms/mycoplasma-genitalium) [20]. Sequence similarity searches were performed using Basic Local Alignment Search Tool for nucleotides (blastn) to identify homologous regions and confirm sequence identities.

### Phylogenetic analysis

Phylogenetic analysis was conducted in a total of 46 genomes including genomes generated in this study and published genomes from Australia (M6320, M6711 and M6270), Europe and Japan (Table S1) [11,21]. Multiple sequence alignments were performed using MAFFT v7.505 [22] with default parameters. Maximum likelihood phylogenetic trees were generated using IQ-TREE v2.2.2.6, and the best-fit nucleotide substitution model was chosen by the ModelFinder algorithm [23]. Tree robustness was estimated using 1,000 bootstrap replicates. *M. genitalium* strain G37 (GenBank accession no. NC_000908) was used as a reference strain and *Mycoplasma pneumoniae* (GenBank accession no. CP010546) as the outgroup. Gubbins v2.2.0 [24] was employed to identify and remove recombinant events within the core-genome alignment.

## Results and discussion

### Culture method to increase bacterial yield

After the initial 2-week establishment of the culture, sampling each week yielded high concentrations of *M. genitalium*, ranging from 10^6^ to 10^8^ geq ml^−1^. DNA extraction from culture aliquots (containing *M. genitalium* loads ranging between 10^7^ and 10^8^ geq ml^−1^) yielded DNA concentrations between 58 and 106 ng µl^−1^ (total DNA yields of 2.9 to 5.3 µg). Moreover, qPCR targeting the *mgpB* gene revealed *M. genitalium* concentrations ranging from 10^6^ to 10^7^ geq µl^−1^. This robust, low-intervention method provided a reliable means for *M. genitalium* propagation, enabling repeated culture sampling and generation of sufficient DNA for comprehensive genomic analyses.

### Sequencing summary

Twenty-two *M. genitalium* cultured isolates were sequenced using the ONT platform, and their genomes were successfully assembled. On average, after filtration by length and read quality, 1.03% of the reads (range: 0.15%–5.40%) mapped to the *M. genitalium* G37 reference genome (NC_000908), resulting in a mean sequencing depth of 51.01× (range: 31.2×–109.0×). The remaining reads were discarded as they aligned to mammalian DNA (range: 94.48%–99.74%) or other contaminants (range: 0.11%–0.12%). The average length of *Mg*-mapped read across the samples was 38,089 bp, ranging from 1,000 to 182,668 bp.

Despite maintaining high Vero cell confluence and obtaining sufficient *M. genitalium* DNA, a high proportion of non-*M. genitalium* reads was obtained. This can be attributed to several factors. First, the inherent nature of cell culture systems involves continuous cellular turnover, resulting in the release of cell-free DNA from Vero cells into the culture medium, even in seemingly healthy, confluent cultures [25]. This process is particularly relevant when culturing fastidious organisms like *M. genitalium*, which require prolonged incubation periods. This phenomenon is exacerbated by the cytotoxic effects of *M. genitalium* on host cells, which may increase cell death and DNA release without visibly affecting confluence [26]. Additionally, the substantial disparity in genome sizes between Vero cells and *M. genitalium* (3 billion bp vs 0.58 million bp) significantly contributes to the high proportion of non-*M. genitalium* sequence reads. Given this vast genomic size difference, even low levels of host DNA contamination can substantially influence sequencing outcomes, particularly in next-generation sequencing.

Further improvements, such as adaptive sequencing, are being investigated to increase the proportion of pathogen-specific reads [9,27]. This approach has demonstrated effective enrichment in both synthetic mock metagenomic communities and complex real samples, reducing the time required to achieve high-accuracy, single-contig assemblies for low-abundance species compared to non-targeted sequencing.

Our analysis suggests that ~2,400 *M*. *genitalium*-specific reads generally resulted in a complete genome assembly with high confidence and accuracy (sequencing depth >30×) (Table 1). However, it is important to note that this is not a rigid threshold, as factors beyond raw read count can influence assembly success. For instance, similar depths with higher read counts were observed in some samples, suggesting that the proportion of on-target reads, rather than total read count alone, plays a significant role in determining assembly quality. The importance of adequate sequencing depth is well-established in bacterial genomics, particularly when employing long-read technologies like ONT. A coverage depth of 30× aligns with findings from Khrenova *et al*. [28], who reported that this level of coverage is generally sufficient for high-quality *de novo* assembly of bacterial genomes using ONT data.

**Table 1.** Description and sequencing metrics for each isolate sequenced

Isolate ID	Specimen	Year of isolation	No. reads mapped to reference	Depth (×)	Genome size (bp)	rRNA operon translocation*
MGA20	HVS	2019	3,927	34.9	581,765	Y
MGA38	HVS	2022	2,403	33.1	581,831	Y
MGA47	HVS	2020	4,648	35.6	581,743	Y
MGA92	Urine	2022	13,148	109.0	582,215	Y
MGA122	HVS	2023	2,601	37.0	581,950	Y
MGA503	Urine	2020	4,836	31.2	581,669	Y
MGA512	Urine	2019	6,758	59.2	579,920	N
MGA600	Urine	2022	5,373	62.1	582,190	Y
MGA649	Urine	2023	6612	95.9	582,145	Y
MGA656	Urine	2023	2,982	46.7	580,096	N
MGA663	Urine	2022	3,207	37.4	582,315	Y
MGA683	Urine	2023	2,529	32.3	580,069	N
MGA710	Urine	2023	3,704	54.3	581,694	Y
MGA719	Urine	2022	2,857	42.8	582,198	Y
MGA720	Urine	2022	5,339	42.0	579,681	N
MGA729	Urine	2023	3,514	40.1	581,709	Y
MGA735	Urine	2022	3,504	41.7	581,712	Y
MGA753	Urine	2023	7,445	81.2	579,659	N
MGA755	Urine	2023	2,957	47.6	579,545	N
MGA759	Urine	2023	4,974	44.5	580,057	N
MGA1014	Urine	2023	3,563	42.4	579,756	N
MGA1026	Urine	2024	6,231	71.1	579,626	N

*Translocation of rRNA region when compared to reference sequence *M. genitalium* G37. Y, yes; N, no. Samples were named with the prefix ‘MAGIC’ and then the participant number, while bacterial isolates were named with the prefix ‘MGA’ (for *M. genitalium*, Australia) followed by the participant number.

HVS, high vaginal swab.

### Genome assembly and annotation

Quantitative genome assessment indicated a high proportion of single-copy orthologues and no evidence of contaminating sequences in all the genomes. The high percentage of complete single-copy universal orthologous genes (BUSCOs) (average 97.3%, range: 96.0%–98.3%) confirmed the completeness of the genome assembly [29]. Genome size ranged from 579,545 to 582,315 bp, with an average GC content of 31.7 mol%.

**Fig. 1.**
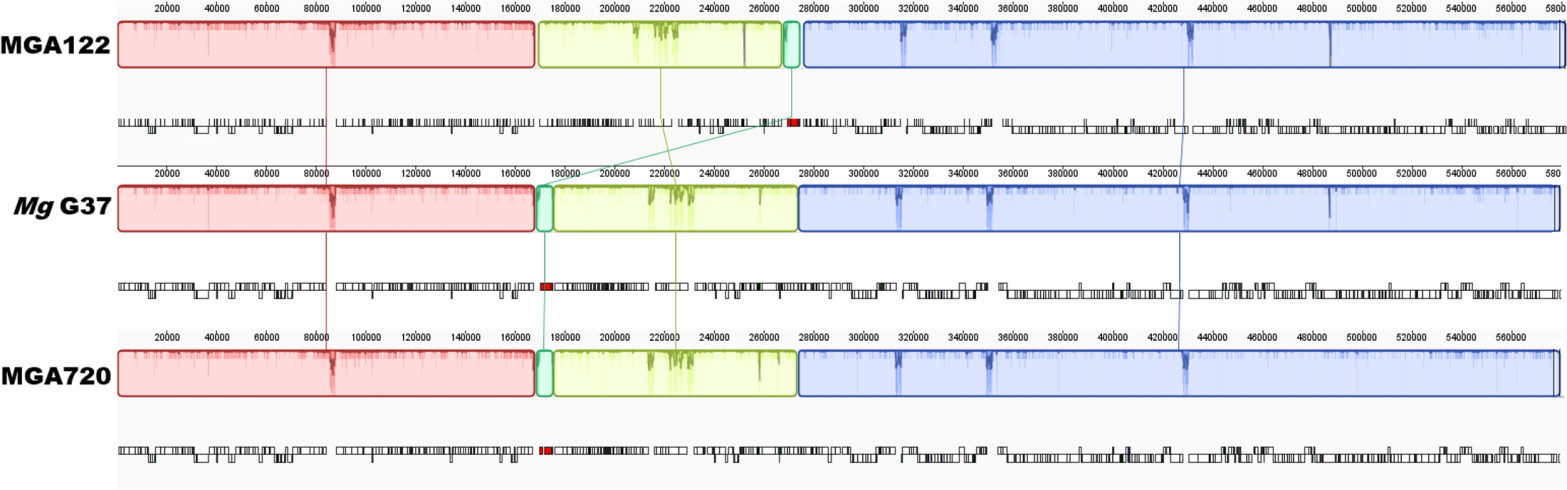
Whole-genome alignment of complete *M. genitalium* genomes using ProgressiveMauve. Coloured blocks represent locally collinear blocks shared among genomes; the green blocks represent rRNA operon that is translocated (compared to the reference genome of G37) in MGA122, representing 13 genomes.

Gene prediction analysis using Geneious Prime identified 525 putative genes per genome, with an average length of 1,029 bp. This included 476 predicted protein-coding sequences, 36 tRNA genes and 3 rRNA genes. Whole-genome alignment of the new Australian *M. genitalium* isolates against the G37 reference strain (NC_000908) was performed using the Mauve progressive alignment algorithm. The analysis resolved the genomes into four locally collinear blocks, representing conserved syntenic segments shared among the isolates and the reference. Most of the genome was organized into large, collinear blocks, indicating high structural conservation across the dataset. However, a translocated genomic segment comprising the rRNA operon (16S, 23S and 5S ribosomal RNA) (Fig. 1) was observed in 59% (13/22) of the new sequences (Table 1). Fig. 1 was constructed using complete genome sequences from isolates displaying translocated and non-translocated genomic arrangements to illustrate this genomic architecture and structural variation. These complete genome sequences encompass both coding and non-coding regions, providing a comprehensive view of the genomic landscape. The translocation of this region was verified by PCR using primer sets mg_75 and mg_78 from Chua *et al*. (2025) [30] (covering positions 167,011-170,549 and 173,846-176,332 of *M. genitalium* G37 respectively), confirming the absence of the locus in the expected position (Fig. S3).

**Fig. 2.**
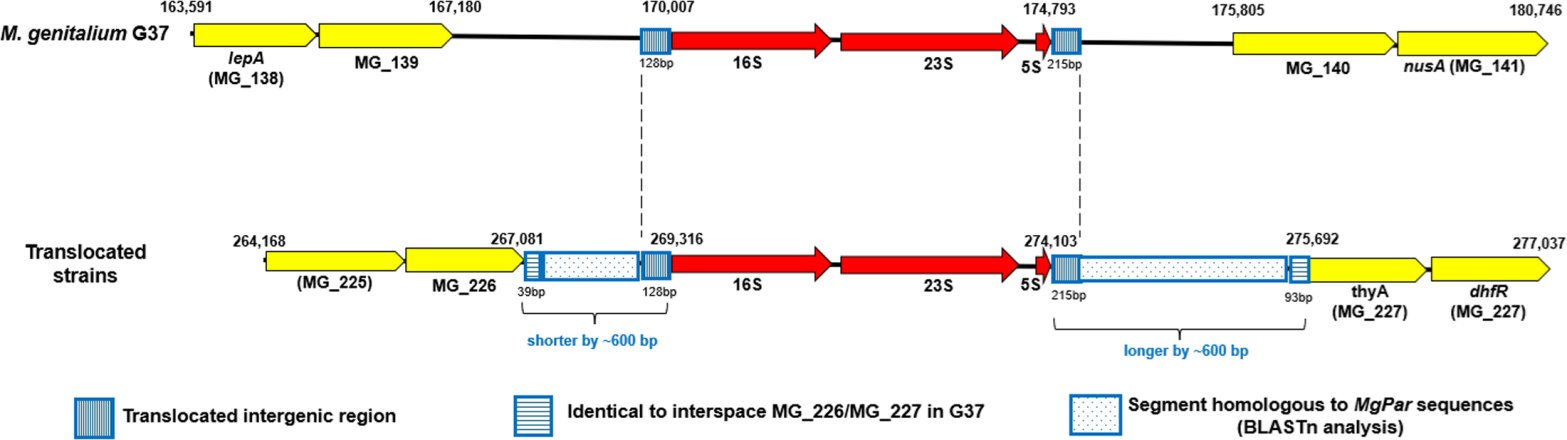
Comparative visualization of genome organizational differences in the standard (G37) genome structure and genome with translocated rRNA operons. Yellow arrows represent protein-coding genes and red arrows indicate rRNA genes. Images adapted from Geneious Prime v2025.0.2.

In reference strain G37, the rRNA operon is located between MG_139 (metallo-beta-lactamase protein) and MG_140 (conserved hypothetical protein), spanning coordinates 170,007 to 174,793 bp. In contrast, in the translocated isolates, the entire operon and adjacent sequences were relocated to a distinct genomic position between MG_226 (amido acid-polyamine organocation permease protein) and MG_227 (*thyA*) (Fig. 2). For the purposes of further comparisons, the G37 arrangement will be referred to as the ‘standard’ genomic structure. In isolates with the translocated operon, the gene orientation was preserved, suggesting a conservative rearrangement rather than gene loss or inversion. Comparative analysis of the rRNA operon, including flanking regions, revealed similar length across all genomes sequenced in this study (range from 8,571 to 8,683 bp). However, translocated strains all displayed a length difference of ~600 bp in the operon flanks compared with reference strain G37 (stippled region, Fig. 2). Specifically, these strains possessed a shorter upstream region adjacent to the 16S rRNA gene (2,229 vs 2,826 bp) and a longer downstream region adjacent to the 5S rRNA gene (1,618 vs 1,011 bp).

These findings indicate that although the rRNA operon coding sequences in *M. genitalium* remain highly conserved, reflecting strong evolutionary constraint, the adjacent intergenic regions display considerable structural flexibility. This pattern aligns with the broader genomic organization described by Fookes *et al*. [11], who demonstrated that *M. genitalium* maintains a compact and conserved core genome punctuated by discrete regions of high variability driven by recombination, particularly within repetitive or surface-exposed regions, such as the *mgpB* gene which undergoes variation for immune evasion [31]. This configuration, characterized by a stable housekeeping backbone and localized genomic plasticity, reflects the evolutionary balance typical of minimal genomes. Foundational mutagenesis work by Glass *et al*. [13] further defined this essential core, identifying only ~100 nonessential genes out of ~480 protein-coding genes. More recently, adaptive evolution of a synthetic minimal cell [32] has shown that even genomes stripped to their essentials retain capacity for compensatory mutations and fitness recovery. Collectively, these studies highlight that *M. genitalium* has reached a point of extreme genomic reduction where essential coding regions, such as the rRNA operon, are preserved under strong selective constraint, while surrounding noncoding sequences act as limited but critical substrates for adaptive flexibility within an otherwise streamlined genome.

Comparative sequence analysis revealed that segments of the upstream and downstream junctions were identical to the corresponding regions in the *M. genitalium* G37 reference genome (stippled region, horizontal striped region, Fig. 2). Moreover, blastn analysis of the flanking sequences identified their closest matches within annotated *MgPar* repetitive regions, demonstrating sequence identity to previously characterized *MgPar* loci rather than to elements associated with known mobile genetic elements. No canonical mobile-element signatures (e.g. transposase ORFs, terminal inverted repeats or long direct repeats) were detected in these segments, arguing against a transposon- or integrative element-mediated mechanism of insertion. Instead, the association with *MgPar* sequences suggests that the observed structural variation resulted from recombination events involving repetitive elements. *MgPar* regions are well established as donor archives that participate in homologous recombination with expressed adhesin genes (*mgpB* and *mgpC*), thereby generating extensive sequence diversity in *M. genitalium* populations [12,33]. The identification of *MgPar*-derived sequences flanking the translocated operon is therefore consistent with a *MgPa*r-mediated recombination mechanism.

### Phylogenetic diversity in Australian *M. genitalium* isolates

Whole-genome comparisons were performed for 46 *M*. *genitalium* strains from Australia, Europe and Asia, including the 22 genomes generated in the present study. Overall, pairwise nucleotide similarities across the dataset ranged between 91.42% (strain MGA92 vs M2300) and 99.94% (strain MGA759 vs G37 reference strain). Among the genomes sequenced in this study, similarities spanned from 96.26% (strain MGA512 vs strain MGA600) to 99.60% (strain MGA20 vs strain MGA735).

**Fig. 3.**
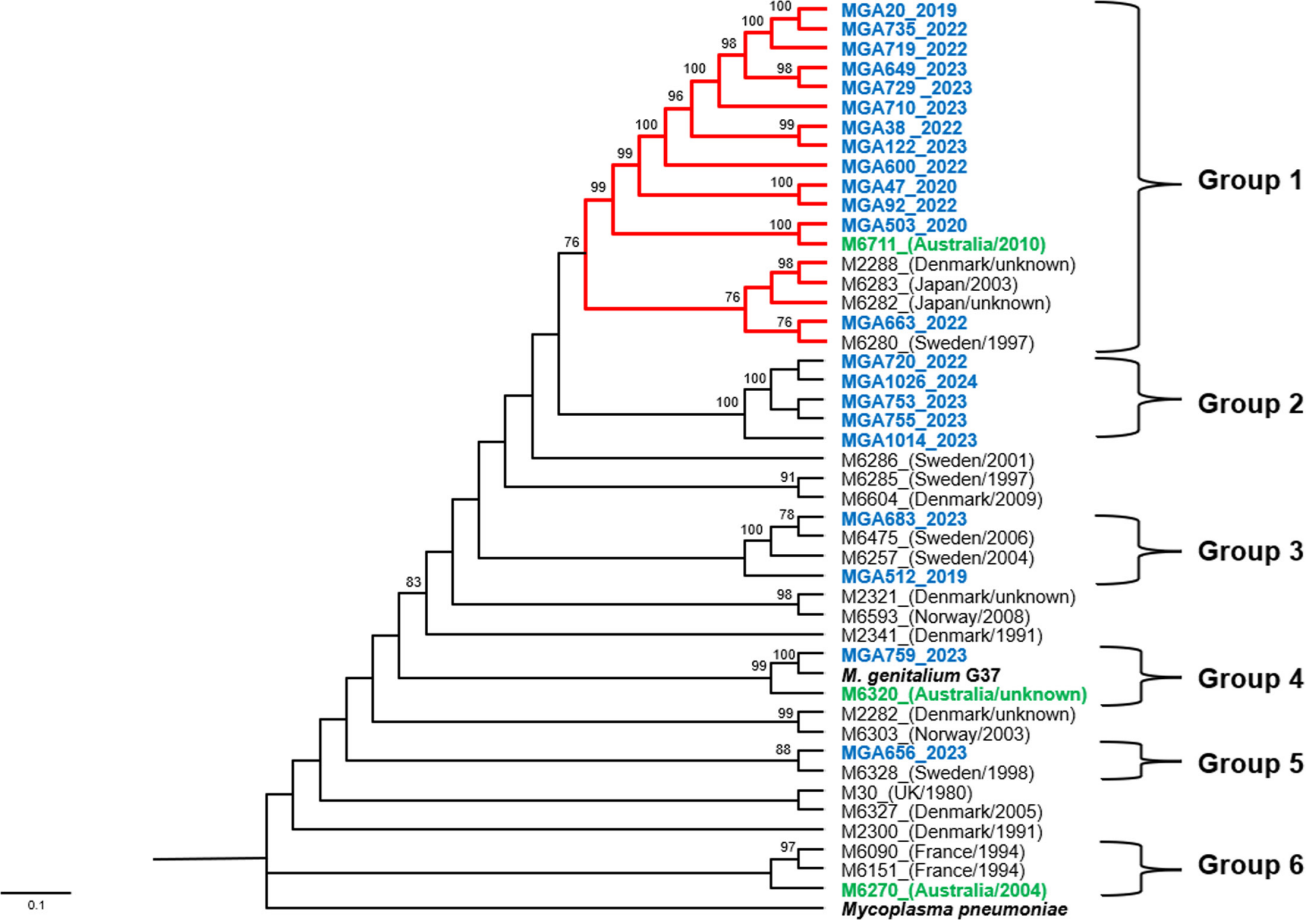
Phylogeny of *M. genitalium* strains based on 46 complete genomes. Genomes generated in this study are shown in blue, other Australian genomes in green and non-Australian genomes in black. Year of isolation and country are indicated. *M. genitalium* G37 (NC_000908) was used as reference strain and *M. pneumoniae* (CP010546) as the outgroup. Branches in red denote samples with translocated rRNA operon. The tree was generated by the GTR+F+I+G4 substitution model. Bootstrap support values are indicated at major nodes; values above 75% are shown.

Australian *M. genitalium* isolates segregated into six groups, reflecting a complex evolutionary history that indicated both diversification and potential transmission events across geographical boundaries (Fig. 3). Groups were defined based on the presence of one or more Australian genomes. Notably, strains harbouring the translocated rRNA operon clustered in a well-supported monophyletic group, corresponding to the red-highlighted clade (Group 1) in Fig. 3. This translocation lineage encompasses strains from diverse geographical locations such as Australia, Denmark, Japan and Sweden, isolated from as far back as 1997. This phylogenetic distribution suggests that the rRNA operon translocation event is present in *M. genitalium* strains across Europe, Asia and Australia. While the close genetic relationship among these isolates implies a shared common ancestor, the temporal span of isolates (from 1997 to present) and their geographical diversity indicate a more gradual dissemination rather than a recent, localized expansion. The genetic distances observed among non-translocated strains across the phylogenetic tree further support a pattern of ongoing evolution and diversification over time, rather than a rapid, recent expansion event.

Phylogenetic analysis was also conducted on genome sequences with the rRNA operon omitted to identify if this was a major factor influencing the tree and group designation (Fig. S1). The quantitative comparison of the phylogenetic trees constructed with and without the rRNA operon yielded both a Robinson–Foulds distance and a quartet distance of 0, indicating identical topologies between the two trees across all levels of comparison, from individual splits to relationships among all sets of four taxa. Moreover, no recombination events were detected in the obtained genomes, and the phylogenetic tree topology exhibited a consistent pattern across all sequences (Fig. S2, also available on Figshare DOI: 10.6084/m9.figshare.31062259). The congruence between these phylogenetic reconstructions suggests that the translocation of the rRNA operon is not a substantial factor in the determination of evolutionary relationships among the studied strains.

Unlike Group 1, isolates with standard genomic structure are positioned on longer branches, indicating deeper evolutionary divergence and higher intra-lineage diversity. Isolates sequenced in this study form distinct and well-supported groups. For instance, Group 2 has strong bootstrap support (100%) and is phylogenetically distinct from other groups and divergent branches. This group likely represents an older lineage that has persisted independently of the recent expansion observed in Group 1. In contrast, other isolates such as MGA683, MGA512 and MGA759 occupy more basal positions, branching off earlier in the tree and contributing to the observed phylogenetic heterogeneity. Interestingly, strain MGA759 and reference strain *M. genitalium* G37 grouped in a strongly supported cluster (Group 4). This close association indicates minimal genetic divergence between these two strains, suggesting that some Australian lineages remain highly conserved relative to the reference strain that was originally isolated around 45 years earlier.

Our genomic analysis, alongside other Australian strains, reveals the concurrent presence of both recently expanded and divergent *M. genitalium* lineages. Notably, two of the three other Australian strains cluster with our isolates (Groups 1 and 4, Fig. 3). M6711 is part of the translocated rRNA operon group, closely associated with most of our isolates. High bootstrap values and short branch lengths suggest recent clonal expansion from a common ancestor. Moreover, our data suggest that these translocated strains have circulated in Australia since at least 2010. In contrast, M6320 and MGA759 align with the reference strain G37 (Group 4), while M6270 (Group 6) belongs to a more divergent lineage, suggesting possible evolutionary divergence among Australian isolates.

Social and demographic factors are likely to contribute to the genetic diversity of *M. genitalium* strains observed in our study. This includes the prominence of Melbourne as a hub for international students and the recent increase in immigration to Australia. Thus, the multicultural population enhances heterogeneity within the circulating *M. genitalium* strains. This interplay of diverse demographics and the high mobility of international students and travellers/backpackers may facilitate both the introduction of novel lineages and their subsequent dissemination. However, detailed demographic information for the individual samples was not available, so these associations could not be examined directly in this study.

Notably, the primary focus of this study was to present methodology for efficient sequencing of *M. genitalium* and to increase the number of available complete genome sequences. We have included limited phylogenetic analysis based on complete genome sequences, and we are currently working on a more detailed analysis comparing phylogeny based on genomes with that using *mgpB*, which is beyond the scope of the current study.

### Prevalence and distribution of antimicrobial resistance markers

In this study, we examined selected antimicrobial resistance (AMR)-related genes to contextualize our genome sequencing results. To maintain the scope of our research, we limited the number of genes included in our analysis. As a result, some potentially significant genes such as L22, *parE* and *gyrB* were not investigated in this current work. These genes, along with others of potential significance, will be examined in a separate study. Analysis of our *M. genitalium* isolates revealed a high prevalence of AMR markers. Point mutations at positions 2058 and 2059 (*E. coli* numbering) within domain V of the 23S rRNA gene have been consistently detected in *M. genitalium* strains from patients with azithromycin treatment failure and are known to confer high-level resistance *in vitro* [4,34, 35]. In our study, 11 (50%) isolates carried a known mutation at position 2058, while 9 (41%) harboured a mutation at position 2059 (Table 2). Among these, A2059G was the most frequent substitution (45%, 9/20), followed by A2058G (35%, 7/20) and A2058T (20%, 4/20). Although our dataset represents a selected group of cultured isolates and is therefore subject to sampling bias, the distribution of 23S rRNA mutations closely mirrors that reported in studies worldwide, where A2058G and A2059G are identified as the predominant mutations associated with azithromycin resistance in *M. genitalium* [34,36,38]. While A2058T remains less common globally, recent surveillance studies from Europe indicate a gradual increase in its prevalence [39,41]. This similar pattern suggests that, despite the limited sample size, the resistance profile observed here in Australia reflects international trends in the epidemiology of macrolide resistance [36,37].

**Table 2.** Distribution of resistance-associated markers and sequence types

Sample ID	*mgpB* ST	23S rRNA*	L4	*parC*	*gyrA*
MGA20	161	A2059G	H69R	S83I	M95I
MGA38	7	A2059G	H69R	WT	WT
MGA47	435	A2058G	WT	WT	WT
MGA92	435	A2058G	WT	WT	WT
MGA122	7	WT	WT	WT	WT
MGA503	5	A2058G	WT	WT	WT
MGA512	105	A2058G	WT	WT	WT
MGA600	7	A2058G	WT	WT	WT
MGA649	184	A2059G	H69R	S83I	WT
MGA656	134	A2058G	WT	WT	WT
MGA663	457	A2059G	WT	WT	WT
MGA683	140	A2058G	WT	S83I	WT
MGA710	7	A2059G	H69R	S83I	WT
MGA719	161	A2059G	H69R	S83I	M95I
MGA720	2	A2058T	WT	WT	WT
MGA729	456	A2059G	H69R	S83I	WT
MGA735	161	A2059G	H69R	S83I	M95I
MGA753	2	A2058T	WT	WT	WT
MGA755	23	A2058T	WT	S83R	WT
MGA759	1	WT	WT	WT	WT
MGA1014	3	A2059G	WT	S83I	D99Y
MGA1026	140	A2058T	WT	S83I	WT

*E. coli numbering.

Fluoroquinolone resistance-associated mutations in *parC* were identified in 45.5% (10/22) of isolates, with the S83I substitution being the most prevalent (40.9%, 9/22). A single isolate harboured the S83R mutation. The S83I mutation is a well-established marker of fluoroquinolone resistance in *M. genitalium*. This mutation is associated with significantly reduced cure rates, with success rates as low as 41.3% [42]. Although less prevalent than the S83I mutation, S83R has been associated with a reduction in the efficacy of fluoroquinolones (~50% moxifloxacin failure rate) [42]. Its low frequency may reflect a fitness cost to the bacterium, which limits its dissemination despite conferring resistance. This effect is not observed in S83I mutants, which appear to maintain bacterial fitness while conferring resistance [43].

Mutations in *gyrA* were less common and were identified in 18.2% (4/22) of isolates. These mutations included M95I (13.6%, 3/22) and D99Y (4.5%, 1/22). Notably, all samples with *gyrA* resistance mutations also presented S83I mutations in *parC*. This observation aligns with recent reports from Australia [5,44, 45], where the *gyrA*-M95I substitution was the most frequently detected *gyrA* mutation, with a high proportion of them found alongside the *parC*-S83I mutation. Infections harbouring dual *parC/gyrA* mutations have been suggested to be twice as likely to fail moxifloxacin compared with *parC* S83I alone, highlighting the importance of including both genes in future resistance assays [5,44].

Notably, all isolates with QRDR mutations also carried macrolide resistance mutations (MRMs). Although our dataset is relatively small and potentially biassed, this finding is consistent with larger studies reporting increasing rates of dual-class resistance in the Western Pacific, including Australia [3,46]. While our findings should therefore be interpreted with caution, they nevertheless reflect the growing clinical challenge posed by multidrug-resistant *M. genitalium*, particularly in settings where sequential macrolide–fluoroquinolone regimens remain standard practice. These observations further support the need for expanded resistance testing panels that incorporate both 23S rRNA and QRDR mutations, as well as continued efforts to evaluate alternative treatment strategies.

It is important to note that these findings are derived exclusively from cultured isolates, which may introduce bias. Culture-based analyses may favour strains that are more readily cultivable, potentially overrepresenting certain genotypes or resistance profiles while underrepresenting others. Therefore, while our data provide insights into the resistance patterns of the isolates studied, they may not fully reflect the true prevalence of AMR mutations in the broader *M. genitalium* population, including strains that are difficult to culture or present at low abundance in clinical samples.

### Co-occurrence of mutations in L4 and 23S rRNA

The H69R mutation (position corresponding to *E. coli* Gly64) in the L4 ribosomal protein was detected in seven isolates (31.8%) (Table 2). Interestingly, all these isolates also carried the A2059G substitution in the 23S rRNA, while six of them also harboured mutations in the fluoroquinolone resistance-associated genes *parC* and/or *gyrA*. The repeated occurrence of the H69R substitution across multiple isolates suggests that it may not represent a random polymorphism.

Mutations in L4 protein have been associated with macrolide resistance by altering the 23S rRNA and modifying the diameter of the ribosomal exit tunnel [47]. The L4 protein contains extended loop regions that converge to form a constriction near the macrolide-binding site, and missense mutations associated with resistance often cluster in this region. These mutations are frequently localized to residues Gln62–Arg67 in *E. coli* (equivalent to Gln67-Lys72 in *M. genitalium*) [48]. *In vitro* selection experiments in *M. pneumoniae* have demonstrated that L4 substitutions such as H70R or H70L can contribute to macrolide resistance [40]. However, the role of L4 mutations in macrolide resistance in *M. genitalium* remains uncertain. L4 mutations in *M. genitalium* are typically located outside the loop region [35,49] and have not been associated with clinical treatment outcomes [49]. Co-occurrence of L4 loop alterations with established 23S rRNA resistance mutations has been rarely documented in *M. genitalium*, with only a single report describing this pattern [50]. In the study by Jensen *et al*. [50], the H69R substitution was identified in four *M. genitalium* strains that had been tested *in vitro* and confirmed as macrolide resistant. All these strains also harboured the A2059G mutation in 23S rRNA, consistent with the pattern observed in our isolates. Importantly, the presence of H69R did not further increase the minimum inhibitory concentration compared with strains carrying only the 23S rRNA mutation [50].

The specific contribution of L4 mutations, such as H69R, to macrolide resistance in *M. genitalium* remains challenging to determine definitively. The consistent co-occurrence of L4 mutations with 23S rRNA mutations in resistant strains complicates the assessment of their independent effects. Additionally, the lack of clinical isolates carrying L4 mutations alone (without 23S rRNA mutations) further hinders our understanding of their specific role in resistance. The limited data available make it difficult to conclude whether L4 mutations like H69R can increase macrolide MICs beyond the levels conferred by 23S rRNA mutations alone.

Future research should focus on conducting comprehensive MIC studies to elucidate the potential modulatory or compensatory role of H69R and other L4 mutations in *M. genitalium*. This could include *in vitro* studies with isogenic mutants to isolate the effects of L4 mutations, as well as longitudinal clinical investigations linking L4 mutations to treatment outcomes. Such studies could help clarify whether these secondary alterations impact the persistence of resistance, bacterial fitness or the development of multidrug-resistant strains, providing a more comprehensive understanding of the complex mechanisms underlying macrolide resistance in *M. genitalium*.

### Association between resistance markers and *mgpB* sequence types

Our analysis identified 14 distinct *mgpB* alleles (ST) including two novel types (456 and 457) which were deposited in the PubMLST database (Tables 2 and S4). ST-7 emerged as the most prevalent allele in our study (18.2%), aligning with previous data from Australia [51], where 225 samples collected between 2017 and 2019 also identified ST-7 as a predominant allele in two Australian states (Victoria and Queensland). This concordance suggests that ST-7 has been stably circulating over time in Australia. Interestingly, this pattern contrasts with observations in Europe, where studies have reported ST-4 as more dominant [52,53]. The disparity in dominant alleles between Australia and Europe could potentially be attributed to several factors including geographical isolation of Australia, varying selection pressures due to antibiotic usage patterns, or temporal changes in strain distribution. Further investigation into these factors could provide valuable insights into the dynamics of *mgpB* allele distribution across diverse populations and time periods.

A marked correlation was observed between *mgpB* ST-161 and a specific dual-class resistance profile. Three isolates (13.6%), MGA20, MGA735 and MGA719, belonged to ST-161 and exhibited an identical resistance genotype, carrying mutations in 23S rRNA (A2059G), L4 (H69R), *parC* (S83I) and *gyrA* (M95I). Notably, they were also the only isolates to harbour the *parC* V701A mutation (Table S3), which, to our knowledge, has not been previously reported. The consistent co-occurrence of multiple resistance mutations with ST-161 highlights a robust association that reinforces the utility of *mgpB* typing for tracking resistant lineages and raises concern about the potential emergence of novel multidrug-resistant clones in Australia.

Our findings suggest a hypothetical link between *mgpB* ST-161, dual-class resistance and the H69R substitution in ribosomal protein L4, but caution is warranted given the limited number of isolates analysed in this study. Further research using larger, demographically and geographically diverse datasets will be required to determine whether ST-161 is linked to a specific sexual network or whether it may serve as a marker of multidrug resistance in *M. genitalium*. Previous studies have shown that certain *mgpB* sequence types are associated with specific sexual networks and resistance profiles [51,53, 54]; for instance, ST-4 and ST-105 have been predominantly detected in men who have sex with men and frequently harbour MRMs [51,55,58]. A key limitation of our study is the relatively small sample size and the absence of detailed epidemiological data on sex and sexual orientation for some isolates and the lack of epidemiological data on sexual orientation. In particular, the lack of sexual orientation data limits our ability to explore potential associations within our dataset.

## Conclusion

This study presents a practical methodology for whole-genome sequencing of *M. genitalium* and contributes 22 new genomes, thereby expanding the currently limited genomic dataset available for future research. The association of dual-class resistance, *mgpB* ST161 and ribosomal protein L4 substitutions highlights a possible emerging clone. Together, these findings underscore the structural plasticity and evolutionary complexity of *M. genitalium* and demonstrate the value of integrated genomic surveillance and resistance detection to inform treatment strategies and identify potential resistant lineages.

## Supplementary material

10.1099/mgen.0.001622 Uncited Supplementary Material 1.

## Abbreviations

AMR: antimicrobial resistance
blastn: Basic Local Alignment Search Tool for nucleotides
gDNA: Genomic DNA
geq: genome equivalents
MRM: macrolide resistance mutation
ONT: Oxford Nanopore Technologies
qPCR: quantitative PCR
QRDR: quinolone resistance-determining region
