# Supplementary material for: Novel features of Mycoplasma genitalium genomes identified through Oxford Nanopore sequence analysis of isolates from Australia
Source: Microb Genom. 2026 Jan 28;12(1):001622. doi: 10.1099/mgen.0.001622 (PMC12852374; doi:10.1099/mgen.0.001622)
Supplement: Uncited Supplementary Material 1. [file mgen-12-01622-s001.pdf]

Supporting Information

Novel features of *Mycoplasma genitalium* genomes identified through Oxford Nanopore sequence analysis of isolates from Australia

Jose L. Huaman[1,2,3], Catriona S. Bradshaw[4,5,6], Teck-Phui Chua[1,2,3], Erica L. Plummer[1,2,4,5], Jennifer A. Danielewski[1,2,3], Lenka A. Vodstrcil[4,5,6], Suzanne M. Garland[1,2,3], Gerald L. Murray[1,2,3]

1. Department of Obstetrics, Gynaecology and Newborn Health, University of Melbourne, Parkville, Victoria, Australia
2. Centre for Women's Infectious Diseases, The Royal Women's Hospital, Parkville, Victoria, Australia
3. Molecular Microbiology Research Group, Murdoch Children's Research Institute, Parkville, Victoria, Australia
4. Melbourne Sexual Health Centre, Alfred Health, Carlton, Victoria, Australia
5. School of Translational Medicine, Monash University, Melbourne, Victoria, Australia
6. Centre for Epidemiology and Biostatistics, Melbourne School of Population and Global Health, University of Melbourne, Parkville, Victoria, Australia

Total pages: 10

Number of figures: 3

Number of Tables: 4

Table S1: Details of sequences included in the phylogenetic analysis

Sequence ID	Database	Accession number
M2282	PubMLST	N/A
M2288	GenBank	NC_018498.1
M2300	PubMLST	N/A
M2321	GenBank	NC_018495.1
M2341	PubMLST	N/A
M30	PubMLST	N/A
M6090	PubMLST	N/A
M6151	PubMLST	N/A
M6257	PubMLST	N/A
M6270	PubMLST	N/A
M6280	PubMLST	N/A
M6282	GenBank	NC_018496.1
M6283	PubMLST	N/A
M6285	PubMLST	N/A
M6286	PubMLST	N/A
M6303	PubMLST	N/A
M6320	GenBank	NC_018497.1
M6327	PubMLST	N/A
M6328	PubMLST	N/A
M6475	PubMLST	N/A
M6593	PubMLST	N/A
M6604	PubMLST	N/A
M6711	PubMLST	N/A

N/A: not applicable

Table S2: Data description submitted to GenBank database

Sample ID	Isolate ID	Collection date	Specimen	genome length (bp)	BioProject	Biosample
MAGIC_20	MGA20	2019	HVS	581,765	PRJNA1326634	SAMN51257948
MAGIC_38	MGA38	2022	HVS	581,831	PRJNA1326634	SAMN51830725
MAGIC_47	MGA47	2020	HVS	581,743	PRJNA1326634	SAMN51830726
MAGIC_92	MGA92	2022	Urine	582,215	PRJNA1326634	SAMN51830727
MAGIC_122	MGA122	2023	HVS	581,950	PRJNA1326634	SAMN51830728
MAGIC_503	MGA503	2020	Urine	581,669	PRJNA1326634	SAMN51830729
MAGIC_512	MGA512	2019	Urine	579,920	PRJNA1326634	SAMN51830730
MAGIC_600	MGA600	2022	Urine	582,190	PRJNA1326634	SAMN51830731
MAGIC_649	MGA649	2023	Urine	582,145	PRJNA1326634	SAMN51830732
MAGIC_656	MGA656	2023	Urine	580,096	PRJNA1326634	SAMN51830733
MAGIC_663	MGA663	2022	Urine	582,315	PRJNA1326634	SAMN51830734
MAGIC_683	MGA683	2023	Urine	580,069	PRJNA1326634	SAMN51830735
MAGIC_710	MGA710	2023	Urine	581,694	PRJNA1326634	SAMN51830736
MAGIC_719	MGA719	2022	Urine	582,198	PRJNA1326634	SAMN51830737
MAGIC_720	MGA720	2022	Urine	579,681	PRJNA1326634	SAMN51830738
MAGIC_729	MGA729	2023	Urine	581,709	PRJNA1326634	SAMN51830739
MAGIC_735	MGA735	2022	Urine	581,712	PRJNA1326634	SAMN51830740
MAGIC_753	MGA753	2023	Urine	579,659	PRJNA1326634	SAMN51830741
MAGIC_755	MGA755	2023	Urine	579,545	PRJNA1326634	SAMN51830742
MAGIC_759	MGA759	2023	Urine	580,057	PRJNA1326634	SAMN51830743
MAGIC_1014	MGA1014	2023	Urine	579,756	PRJNA1326634	SAMN51830744
MAGIC_1026	MGA1026	2024	Urine	579,626	PRJNA1326634	SAMN51830745

HVS: High vaginal swab

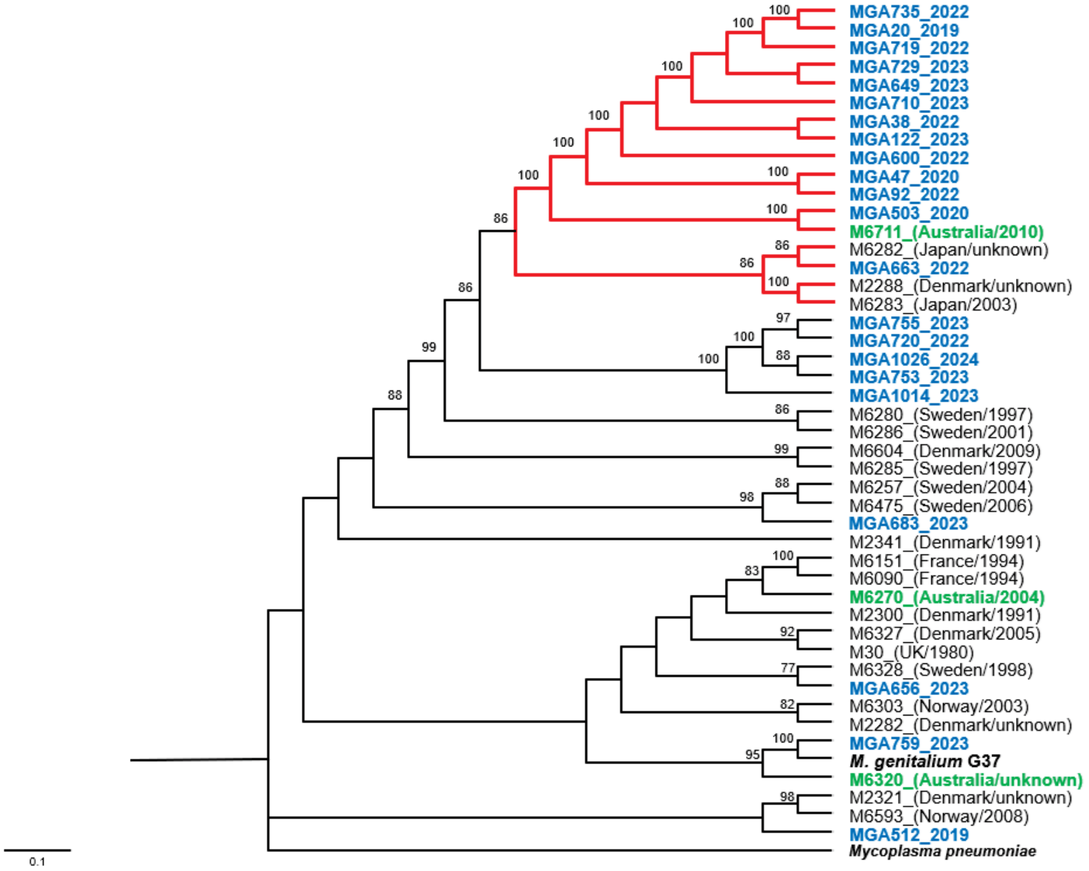

Figure S1. Phylogeny of *M. genitalium* strains based on complete genome sequences with the rRNA operon and flanking regions omitted. Genomes obtained in this study are shown in blue, other Australian genomes in green, and non-Australian genomes in black. *M. genitalium* G37 (NC_000908) was used as reference strain and *M. pneumoniae* (CP010546) as outgroup. Branches in red denote samples with translocated rRNA operon. The tree was generated by the GTR+F+I+G4 substitution model. Bootstrap support values are indicated at major nodes; values above 75% are shown.

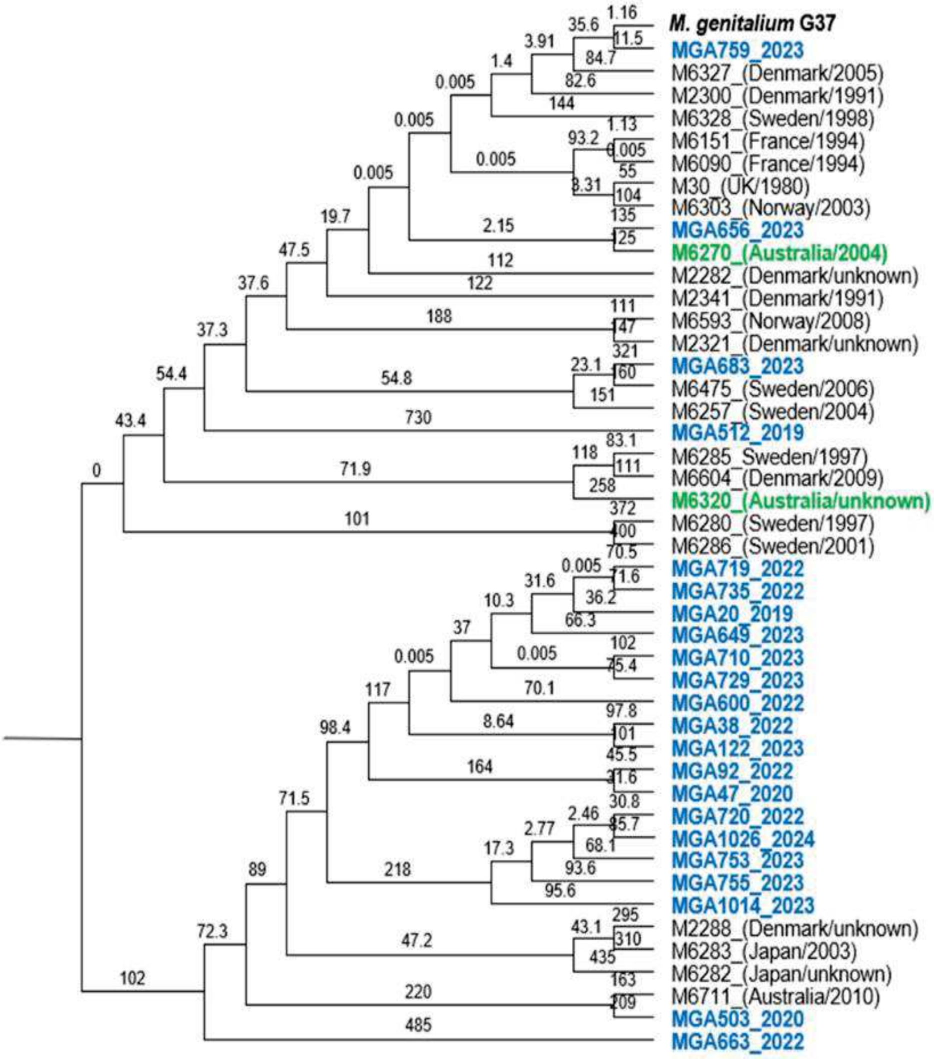

Figure S2. Phylogeny of *M. genitalium* strains based on complete genome sequences using Gubbins. Genomes obtained in this study are shown in blue, other Australian genomes in green, and non-Australian genomes in black. *M. genitalium* G37 (NC_000908) was used as reference strain. Branches in red denote samples with translocated rRNA operon. Numbers in the tree indicates number of substitutions per site.

Table S3. List of amino acid changes identified in *parC*, gyrA and L4 sequences from isolates sequenced in this study. Mutations at position 701 of *parC* are highlighted in yellow.

		Isolate ID																					
	aa change	MGA20	MGA38	MGA47	MGA92	MGA122	MGA503	MGA512	MGA600	MGA649	MGA656	MGA663	MGA683	MGA710	MGA719	MGA720	MGA729	MGA735	MGA753	MGA755	MGA759	MGA1014	MGA1026
parC	S83I	X								X			X	X	X		X	X				X	X
	S83R																			X			
	F225S														X								
	V270I	X	X			X			X	X				X	X		X	X			X		
	H349Y	X	X			X			X	X				X	X		X	X			X		
	M594I	X	X			X			X	X				X	X		X	X			X		
	L602F							X															
	H637Q	X	X			X			X	X			X	X	X	X	X	X	X	X	X		X
	H637Y				X																		
	K673E					X																	
	V701A	X													X			X					
	Q714R										X												
gyrA	G93C												X										
	M95I	X													X			X					
	D99Y																					X	
	A300D												X										
L4	K66E									X													
	H69R	X	X							X				X	X		X	X					
	P81S																						
	L119F										X												
	M142I														X								

Table S4. List of sequences used for genotyping *mgpB* ST.

Sample ID	***mgpB* ST**	Sequence
MGA20	161	ACTTGAAACAATAACAACTTCTCTTCACTAAAGATTACTGGAGAGAACCCAGGATCATTTGGACTAGTAAGAAGTCAAAATGAGAACTTAAACATC GCAAGTGTTACAAAGAATGGTAGTGATGATAATCTCAAGTATCTCAATGCTGTTGAGAAATACCTTGATGGTCAGCAAAACTTTGCAATCAGAAGGT ATGATAACAACGGTAGAGCTTTATATGATATTAACTTA
MGA38	7	ACTTGAAACAATAACAACTTCTCTTCACTAAAGATTACTGGAGAGAACCCAGGATCATTTGGACTAGTAAGAAGCCAAAATGAGAACTTAAACATC GCAAGTGTTACAAAGAATGGTAGTGATGATAATCTCAAGTATCTCAATGCTGTTGAGAAATACCTTGATGGTCAGCAAAACTTTGCAATCAGAAGGT ATGATAACAACGGTAGAGCTTTATATGATATTAACTTA
MGA47	435	ACTTGAAACAATAACAACTTCTCTTCACTAAAGATTACTGGAGAGAACCCAGGATCATTTGGATTAGTAAGAAGCCAAAATGACAACTTAAATATTT CAAGTGTTACAAAGAATGGTAGTGATGATAATCTCAAGTATCTCAATGATGTTGAGAAATACCTTGATGGTCAGCAAAACTTTGCAATCAGAAGGTAT GATAACAACGGTAGAGCTTTATATGATATTAACTTA
MGA92	435	ACTTGAAACAATAACAACTTCTCTTCACTAAAGATTACTGGAGAGAACCCAGGATCATTTGGATTAGTAAGAAGCCAAAATGACAACTTAAATATTT CAAGTGTTACAAAGAATGGTAGTGATGATAATCTCAAGTATCTCAATGATGTTGAGAAATACCTTGATGGTCAGCAAAACTTTGCAATCAGAAGGTAT GATAACAACGGTAGAGCTTTATATGATATTAACTTA
MGA122	7	ACTTGAAACAATAACAACTTCTCTTCACTAAAGATTACTGGAGAGAACCCAGGATCATTTGGACTAGTAAGAAGCCAAAATGAGAACTTAAACATC GCAAGTGTTACAAAGAATGGTAGTGATGATAATCTCAAGTATCTCAATGCTGTTGAGAAATACCTTGATGGTCAGCAAAACTTTGCAATCAGAAGGT ATGATAACAACGGTAGAGCTTTATATGATATTAACTTA
MGA503	5	ACTTGAAACAATAACAACTTCTCTTCACTAAAGATTACTGGAGAGAACCCAGGATCATTTGGATTAGTAAGAAGCCAAAATGACAACTTAAATATTT CAAGTGTTACAAAGAATGTTAGTGATGATAATCTCAAGTATCTCAATGATGTTGAGAAATACCTTGATGGTCAGCAAAACTTTGCAATCAGAAGGTAT GATAACAACGGTAGAGCTTTATATGATATTAACTTA
MGA512	105	ACTTGAAACAATAACAACTTCTCTTCACTGAAGATTACTGGAGAGAACCCAGGATCATTTGGACTAGTAAGAAGCCAAAATGAGAACTTAAACATC GCAAGTGTTACAAAGAATGGTAGTGATGATAATCTCAAGTATCTTAATGCTGTTGAGAAATACCTTGATGGTCAGCAAAACTTTGCAATCAGAAGGT ATGATAACAACGGTAGAGCTTTATATGATATTAACTTA
MGA600	7	ACTTGAAACAATAACAACTTCTCTTCACTAAAGATTACTGGAGAGAACCCAGGATCATTTGGACTAGTAAGAAGCCAAAATGAGAACTTAAACATC GCAAGTGTTACAAAGAATGGTAGTGATGATAATCTCAAGTATCTCAATGCTGTTGAGAAATACCTTGATGGTCAGCAAAACTTTGCAATCAGAAGGT ATGATAACAACGGTAGAGCTTTATATGATATTAACTTA
MGA649	184	ACTTGAAACAATAACAACTTCTCTTCACTAAAGATTACTGGAGAGAACCCAGGATCATTTGGACTAGTAAGAAGTCAAAATGAGAACTTAAACATC GCAAGTGTTACAAAGAATGGTAGTGATGATAATCTCAAGTATCTCAATGCTGTTGAGAAATACCTTGATGATCAGCAAAACTTTGCAATCAGAAGGT ATGATAACAACGGTAGAGCTTTATATGATATTAACTTA

MGA656	134	ACTTGAAACAATAACAACTTCTCTTCACTGAAGATTACTGGAGAGAACCCAGGATCATTTGGACTAGTAAAAAGCCAAAATGAGAACTTAAACATC GCAAGTGTTACAAAGAATGATAGTGATGATAATCTCAAGTATCTTAATTCTGTTGAGAAATACCTTGATGGTCAGCAAAACTTTGCAATCAGAAGGTA TGATAACAACGGTAGAGCTTTATATGATATTAACTTA
MGA663	457	ACTTGAAACAATAACAACTTCTCTTCACTGAAGATTACTGGAGAGAACCCAGGATCATTTGGACTAGTAAGAAGCCAAAATGAGAACTTAAACATC GCAAGTGTTACAAAGAATGGTAGTGATGATAATCTCAAGTATCTTAATGATGTTGAGAAGTACCTTGATGATCAGCAAAACTTTGCAATCAGAAGGTA TGATAACAACGGTAGAGCTTTATATGATATTAACTTA
MGA683	140	ACTTGAAACAATAACAACTTCTCTTCACTAAAGATTACTGGAGAGAACCCAGGATCATTTGGATTAGTAAGAAGCCAAAATGACAACTTAAATATTT CAAGTGTTACAAAGAATGTTAGTGATGATAATCTCAAGTATCTCAATGTTGTTGAGAAATACCTTGATGGTCAGCAAAACTTTGCAATCAGAAGGTAT GATAACAACGGTAGAGCTTTATATGATATTAACTTA
MGA710	7	ACTTGAAACAATAACAACTTCTCTTCACTAAAGATTACTGGAGAGAACCCAGGATCATTTGGACTAGTAAGAAGCCAAAATGAGAACTTAAACATC GCAAGTGTTACAAAGAATGGTAGTGATGATAATCTCAAGTATCTCAATGCTGTTGAGAAATACCTTGATGGTCAGCAAAACTTTGCAATCAGAAGGT ATGATAACAACGGTAGAGCTTTATATGATATTAACTTA
MGA719	161	ACTTGAAACAATAACAACTTCTCTTCACTAAAGATTACTGGAGAGAACCCAGGATCATTTGGACTAGTAAGAAGTCAAAATGAGAACTTAAACATC GCAAGTGTTACAAAGAATGGTAGTGATGATAATCTCAAGTATCTCAATGCTGTTGAGAAATACCTTGATGGTCAGCAAAACTTTGCAATCAGAAGGT ATGATAACAACGGTAGAGCTTTATATGATATTAACTTA
MGA720	2	ACTTGAAACAATAACAACTTCTCTTCACTAAAGATTACTGGAGAGAACCCAGGATCATTTGGATTAGTAAGAAGCCAAAATGACAACTTAAATATTT CAAGTGTTACAAAGAATGTTAGTGATGATAATCTCAAGTATCTCAATGCTGTTGAGAAATACCTTGATGGTCAGCAAAACTTTGCAATCAGAAGGTAT GATAACAACGGTAGAGCTTTATATGATATTAACTTA
MGA729	456	ACTTGAAACAATAACAACTTCTCTTCACTAAAGATTACTGGAGAGAACCCAGGATCATTTGGACTAGTAAGAAGTCAAAATGAGAACTTAAACATC GCAAGTGTTACAAAGAATGGTAGTGATGATAATCTCAAGTATCTCAATGCTGTTGAGAAATACCTTGATGGTAAGCAAAACTTTGCAATCAGAAGGT ATGATAACAACGGTAGAGCTTTATATGATATTAACTTA
MGA735	161	ACTTGAAACAATAACAACTTCTCTTCACTAAAGATTACTGGAGAGAACCCAGGATCATTTGGACTAGTAAGAAGTCAAAATGAGAACTTAAACATC GCAAGTGTTACAAAGAATGGTAGTGATGATAATCTCAAGTATCTCAATGCTGTTGAGAAATACCTTGATGGTCAGCAAAACTTTGCAATCAGAAGGT ATGATAACAACGGTAGAGCTTTATATGATATTAACTTA
MGA753	2	ACTTGAAACAATAACAACTTCTCTTCACTAAAGATTACTGGAGAGAACCCAGGATCATTTGGATTAGTAAGAAGCCAAAATGACAACTTAAATATTT CAAGTGTTACAAAGAATGTTAGTGATGATAATCTCAAGTATCTCAATGCTGTTGAGAAATACCTTGATGGTCAGCAAAACTTTGCAATCAGAAGGTAT GATAACAACGGTAGAGCTTTATATGATATTAACTTA
MGA755	23	ACTTGAAACAATAACAACTTCTCTTCACTAAAGATTACTGGAGAGAACCCAGGATCATTTGGATTAGTAAGAAGCCAAAATGACAACTTAAATATTT CAAGTGTTACAAAGAGTGTTAGTGATGATAATCTCAAGTATCTCAATGCTGTTGAGAAATACCTTGATGGTCAGCAAAACTTTGCAATCAGAAGGTAT GATAACAACGGTAGAGCTTTATATGATATTAACTTA
MGA759	1	ACTTGAAACAATAACAACTTCTCTTCACTAAAGATTACTGGAGAGAACCCAGGATCATTTGGATTAGTAAGAAGCCAAAATGACAACTTAAATATTT CAAGTGTTACAAAGAATTCTAGTGATGATAATCTCAAGTATCTCAATGCTGTTGAGAAATACCTTGATGGTCAGCAAAACTTTGCAATCAGAAGGTAT GATAACAACGGTAGAGCTTTATATGATATTAACTTA

MGA1014	3	ACTTGAAACAATAACAACTTCTCTTCACTGAAGATTACTGGAGAGAACCCAGGATCATTTGGACTAGTAAGAAGCCAAAATGAGAACTTAAACATCGCAAGTGTTACAAAGAATGGTAGTGATGATAATCTCAAGTATCTTAATGCTGTTGAGAAGTACCTTGATGGTCAGCAAAACTTTGCAATCAGAAGGTATGATAACAACGGTAGAGCTTTATATGATATTAACTTA
MGA1026	140	ACTTGAAACAATAACAACTTCTCTTCACTAAAGATTACTGGAGAGAACCCAGGATCATTTGGATTAGTAAGAAGCCAAAATGACAACTTAAATATTTCAAGTGTTACAAAGAATGTTAGTGATGATAATCTCAAGTATCTCAATGTTGTTGAGAAATACCTTGATGGTCAGCAAAACTTTGCAATCAGAAGGTATGATAACAACGGTAGAGCTTTATATGATATTAACTTA

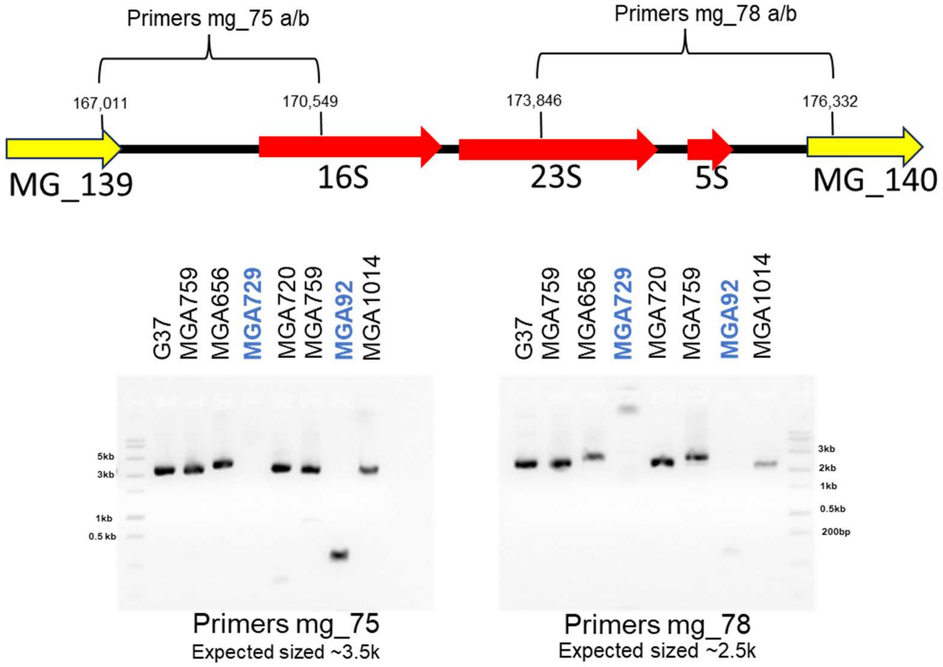

Figure S3. Analysis of the rRNA locus in G37 and other strains. PCR was performed across the junctions of the locus to confirm movement of the region (primers described by Chua et al, 2025). Samples with translocated rRNA operon are highlighted in blue.
